# Disseminated Nocardiosis with subretinal abscess in a patient with nephrotic syndrome-a case report

**DOI:** 10.1186/s12886-018-0883-2

**Published:** 2018-09-03

**Authors:** He Xu, Bo Fu, Li Xu, Jing Sun

**Affiliations:** 1 Department of Ophthalmology, The 4th People’s Hospital of Shenyang, Shenyang, Liaoning People’s Republic of China; 2 Departments of Gastroenterology, The First Affiliated Hospital of China Medical University, Shenyang, Liaoning People’s Republic of China

**Keywords:** Nocardiosis, Subretinal abscess, Endogenous endophthalmitis, Immunosuppression, Nephrotic syndrome

## Abstract

**Background:**

*Nocardia* infection is uncommon in clinical practice, with most cases occuring as the result of opportunistic infection in immunocompromsed patients. Here, we report a case of disseminated nocardiosis with subretinal abscess in a patient with nephrotic syndrome, and whom is receiving immunosuppressive therapy.

**Case presentation:**

A 58-year-old male presented with decreased vision in his left eye, without redness or floaters, which had persisted for three days. The patient had previously been diagnosed with membranous nephropathy, and as such, had received systemic corticosteroid therapy for four months. Further, the patient had developed pneumonia three weeks prior to this presentation. The ocular lesion appeared as a creamy-white subretinal abscess, with overlying retinal hemorrhages. Subsequent administration of three intravitreal injections of vancomycin and ceftazidime ultimately led to eradication of the intraocular infection, however, two months later, the patient developed a brain abcess. Pathogens isolated from the blood were subsequently identified as *Nocardia*. The patient was successfully treated via systemic administration of imipenem and trimethoprim-sulfamethoxazole.

**Conclusions:**

Clinicians should be aware of the possibility of *Nocardia* infections within all immunocompromised patients, as well as the tendency of this infection to disseminate--particularly in the brain. The early detection of *Nocardia* infections and prolonged treatment of the proper antibiotics may significantly improve the prognosis of this life-threatening infection.

## Background

*Nocardia* infection is uncommon in clinical practice, with most cases occuring as the result of opportunistic infection in immunocompromsed patients. Disseminated nocardiosis is a devastating multi-system disease, including brain, eye, joint, kidney, or other organs and tissues involvement. Recent reports have suggested that the incidence of nocardiosis is increasing [1–3]. Herein, we present a case of disseminated nocardiosis with subretinal abscess within a nephrotic syndrome patient receiving immunosuppressive therapy.

## Case presentation

A 58-year-old male presented with decreased vision in his left eye, without redness or floaters, which had persisted for three days. His medical history detailed a diagnosis of nephrotic syndrome (membranous nephropathy), which had been treated with prednisone for a period of four months. Drug-induced diabetes was subsequently detected one month after the onset of corticosteroid treatment. The patient developed productive cough, pyrexia, and night sweats, and was further diagnosed with pneumonia three weeks before the admission. His medications at the time included prednisone 20 mg daily, insulin and oral cefdinir. He had no history of ocular trauma or surgery.

**Fig. 1:**
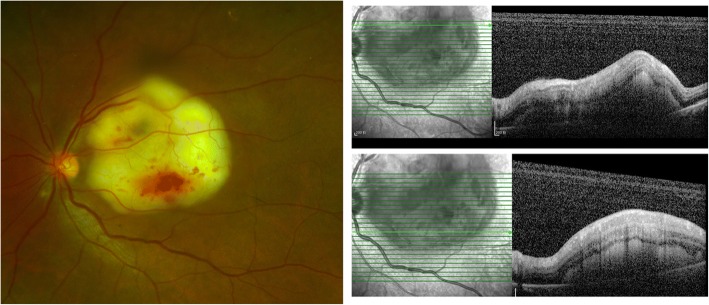
Wide field optomap fundus image and OCT of left eye at presentation. Wide field optomap fundus image showing a creamy-white and raised subretinal lesion at the posterior pole. The lesion was five disc diameters in size and multiple retinal hemorrhages were seen in the overlying retina (left); OCT demonstrated a hyperreflective substance located in the subretinal space, between the outer retina and retinal pigment epithelium (right)

**Fig. 2:**
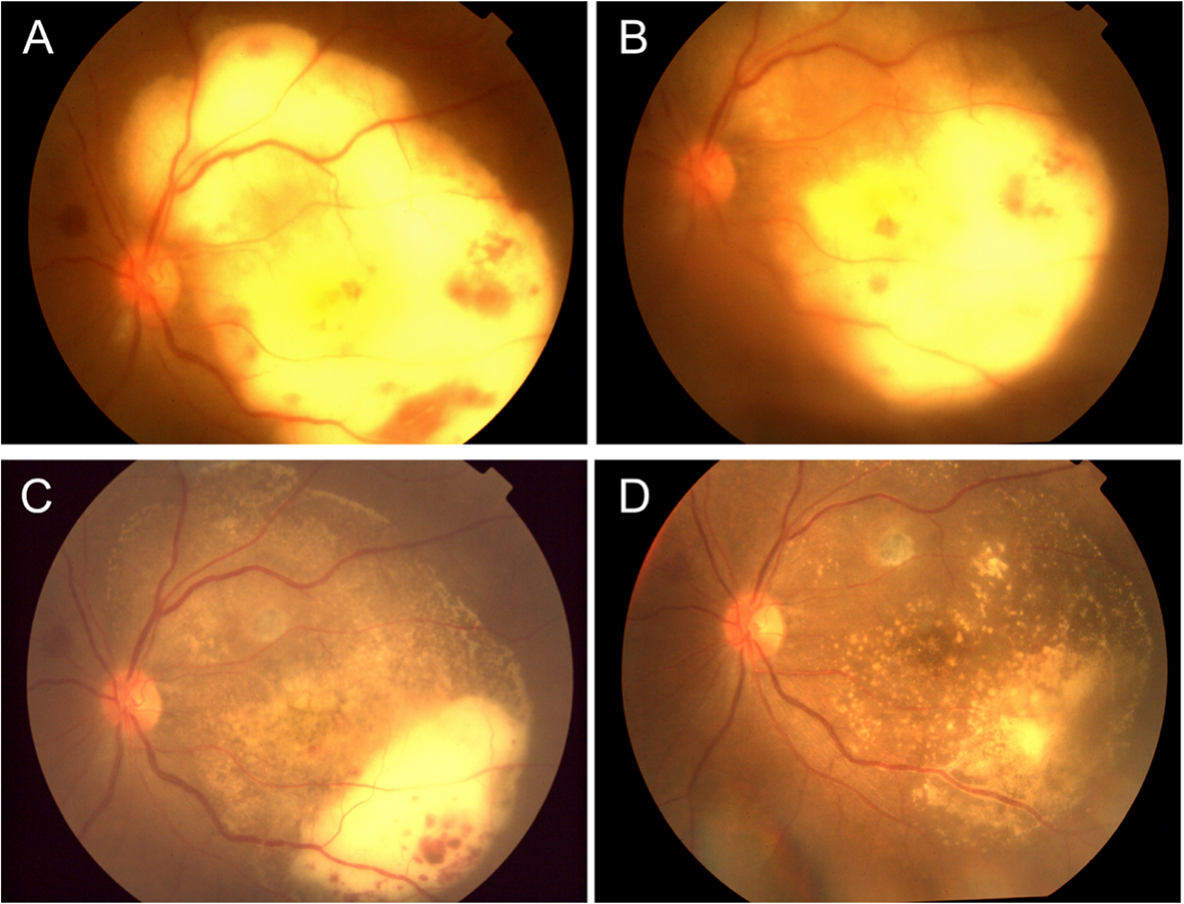
Fundus photographs taken before and after intravitreal injections. Fundus photograph showing mild vitritis and the area of the creamy-white mass had expanded over the superior and inferior arcades at the day of hospitalization (**a**). Three days after initial intravitreal injections, the subretinal abscess had improved significantly (**b**). A week after the second intravitreal injections, the subretinal abscess was gradually relieved and only left small part inferior the lesion (**c**). A month after the third intravitreal injections, the subretinal abscess had resolved, leaving some yellowish subretinal precipitates and chorioretinal scarring (**d**)

An ophthalmic examination revealed visual acuities of 20/20 in the right eye and counting fingers at 2 ft in the left. Pupils were 3 mm and reactive in each eye without relative afferent pupillary defect. The right eye was normal. Slit lamp examination of the left eye was unremarkable. Fundus examination showed clear media and a creamy-white and raised subretinal lesion located at the posterior pole, with the lesion being five disc diameters in size. Additionally, multiple retinal hemorrhages were seen in the overlying retina (Fig. 1). The intraocular pressures were normal in both eyes. Optical coherence tomography (OCT) demonstrated a hyperreflective substance located in the subretinal space, between the outer retina and retinal pigment epithelium (Fig. 1). Although a further diagnostic workup was requested on the follow day of presentation, the patient was lost to follow-up and presented five days later with reduced vision in the affected eye. On examination, slit lamp examination of the left eye showed mild conjunctival injection and anterior chamber cells of 2+. Fundus examination revealed mild vitritis and further, the area of the retinal lesion had expanded over the superior and inferior arcades (Fig. 2a).

**Fig. 3:**
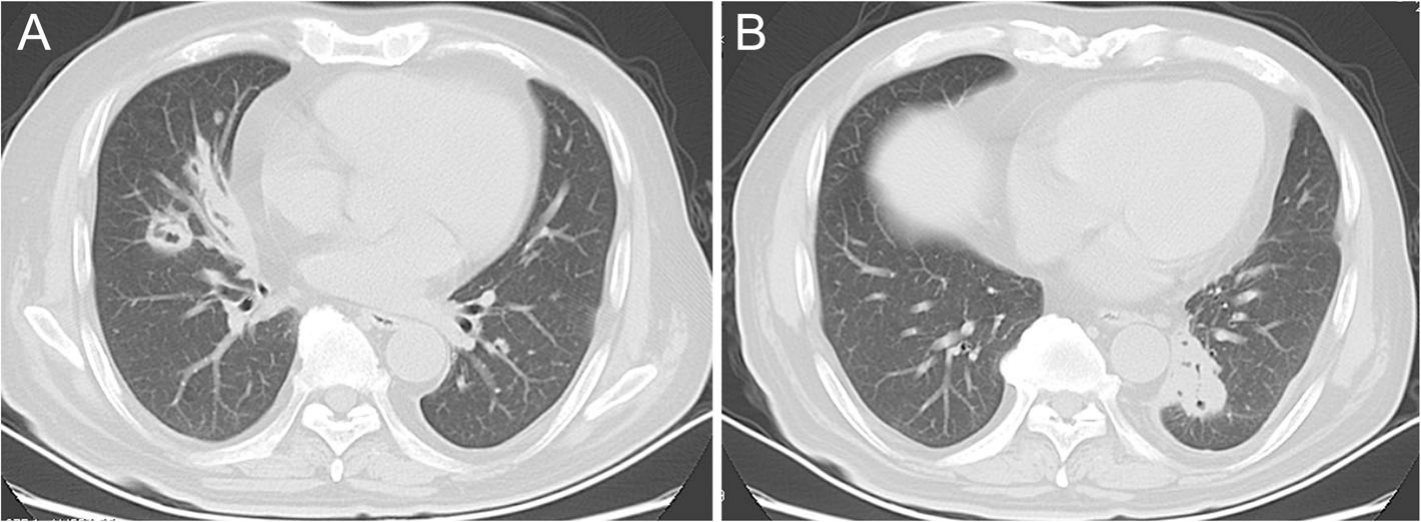
A chest computed tomography scan showing multiple bilateral nodules (**a**, **b**), some of them cavitated in the lungs.

The patient was admitted for further workup. All vital signs were stable and within normal range. The white cell count was 11.48 × 10^9^/l, with 75.8% neutrophils, and the blood level of C-reactive protein was 59.9 mg/liter. Serum markers of human immunodeficiency virus (HIV), syphilis and tuberculosis were all negative. A chest computed tomography (CT) scan showed multiple bilateral nodules, some of them cavitated in the lungs, which suggested of multiple abscesses (Fig. 3). In the context of his lung abscess, a presumptive diagnosis of endogenous endophthalmitis with subretinal abscess was made.

A vitreous tap along with empirical intravitreal injections of a combination of vancomycin (1 mg/0.1 ml) and ceftazidime (2 mg/0.1 ml) were performed on the day of hospitalization. The vitreous samples were sent for bacterial and fungal cultures, as well as bacterial, fungal and viral polymerase chain reaction (PCR). Blood and sputum cultures were also performed. Topical levofloxacin 0.5% and prednisolone acetate 1% eye drops were administrated four times daily, and in addition, oral prednisone and cefdinir were continued. Three days after initial intravitreal injections, the subretinal abscess had improved significantly (Fig. 2b) and a second injection was administered. The subretinal abscess gradually improved, leaving only a small inferior lesion a week later (Fig. 2c). Vitreous cultures and PCR were all negative, as were blood cultures. *Nocardia*, *Heamophilus* and *Candida albicans* were identified in sputum cultures. Based on the clinical data and the characteristic appearance of the ocular lesion, it was deduced that the causative organism was most likely *Nocardia*. As a result, a combination of oral trimethoprim and sulfamethoxazole (TMP-SMX) was administrated. Ten days after hospitalization, the third intravitreal injections were repeated, with the patient then being discharged for follow-up as an outpatient with directions to continue with oral prednisone 20 mg daily and oral TMP-SMX for an further month. The patient remained in stable condition over the following four weeks after discharge. Further fundus examination revealed the subretinal abscess had resolved, leaving some yellowish subretinal precipitates and chorioretinal scarring, with visual acuity of 1/10 (Fig. 2d).

**Fig. 4:**
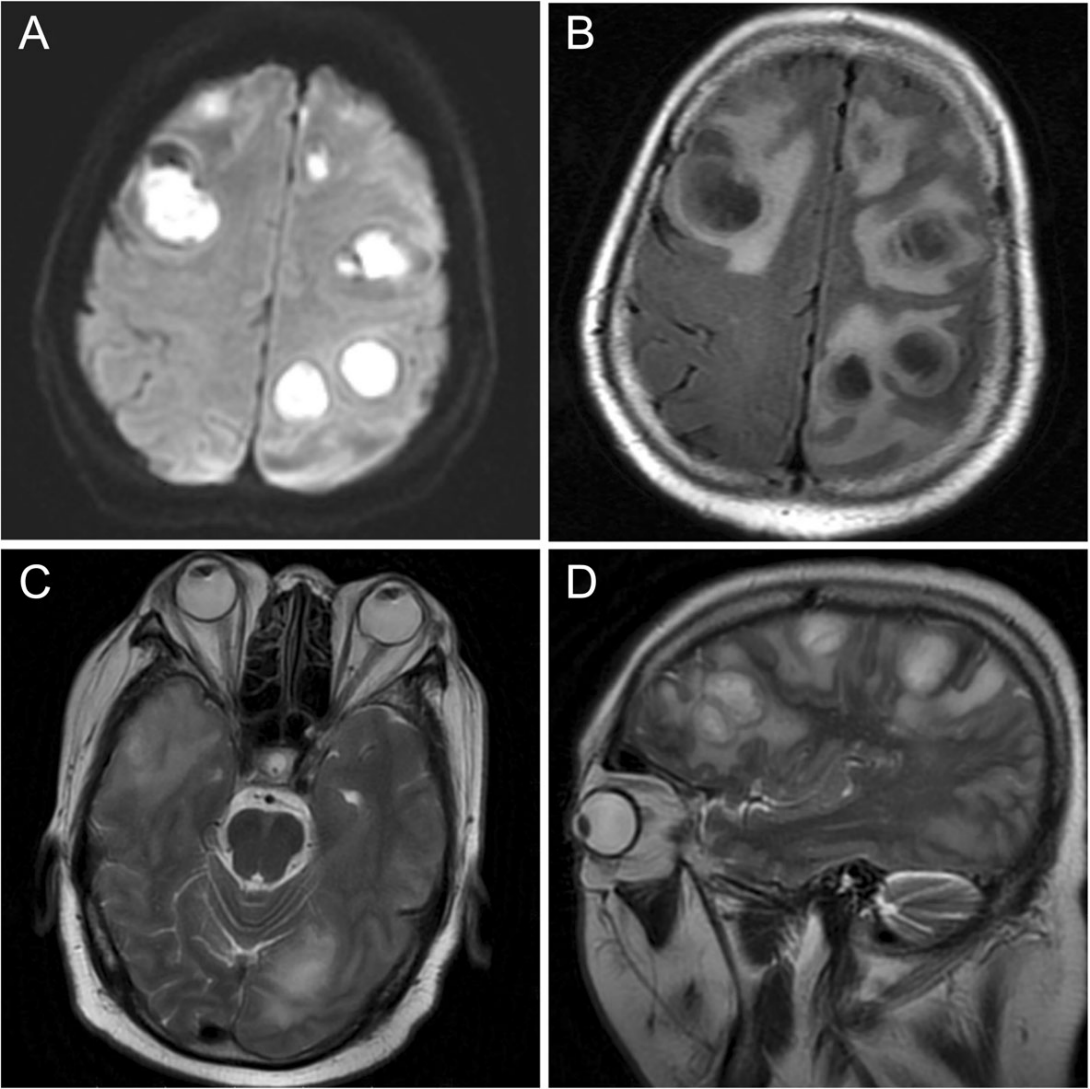
Magnetic resonance images of brain and orbit, showing multiple cystic-enhancing lesions with surrounding edema over both cerebral hemispheres (**a**, **b**, **d**), whilst the eye remained quiescent (**c**, **d**)

Seven weeks after discharge, that is three weeks after cessation of TMP-SMX therapy, this patient was admitted to another hospital due to right extremity weakness and a lack of ability to walk or stand on his own for ten days. Magnetic resonance imaging (MRI) of the brain showed multiple cystic-enhancing lesions with surrounding edema over both cerebral hemispheres, suggestive of multiple abscesses, whilst the retina remained quiescent (Fig. 4). A chest CT scan showed multiple lung abscesses. Blood cultures were positive for *Nocardia*, and as such disseminated nocardiosis was diagnosed. Based on sensitivity data, the patient was administered with intravenous imipenem/cilastatin and oral TMP-SMX, with intravenous therapy being continued for three weeks, followed by TMP-SMX monotherapy. The patient was discharged due to improvement in his systemic condition with the recommendation of close follow-up by an infectious disease specialist. Long-term oral TMP-SMX was also prescribed for the patient. Within follow-up chest CT and brain MRI carried out three months later, it was found that the previously detected foci had become smaller or had completely disappeared. A six-month follow-up indicated that the lung abscesses disappeared, and a brain MRI showed the previous residual lesions had almost completely resolved.

## Discussion

*Nocardia* is a genus of aerobic, gram positive, filamentous, branching bacteria that can be found in soil or water. The organism can infect humans via respiratory inhalation or skin traumatic injury. *Nocardia* has the potential to disseminate hematogenously into various parts of the body, especially the brain, and has a tendency to relapse despite the use of an appropriate therapy [4, 5]. Acute or chronic suppurative necrosis and abscess formation are the pathologic hallmarks of Nocardiosis. Ocular nocardiosis is exceedingly rare, and usually presents with insidious, painless vision loss caused by chorioretinal infiltrates in the macular region. As seen in this case report, ocular nocardiosis initially presented as chorioretinitis with creamy-white subretinal abscess, with subsequent involvement of the vitreous and anterior chamber, and showed a mild inflammatory reaction as the patient’s condition deterioriated.

Treatment of ocular *Nocardia* infections will vary with the severity and location of the disease. Generally, systemic antibiotic treatment is considered the cornerstone therapy for endogenous bacterial endophthalmitis, but this approach may bring about inadequate therapeutic levels of drug within the intraocular spaces. Treatment with intravitreal antibiotics has been suggested to provide high local concentration of the drug, however, a more aggressive infection will require surgical intervention and systemic therapy. Previous reports have described poor outcomes of ocular *Nocardia* infections that often resulted in blindness [6]. But in this case, three intravitreal injections have led to eradication of the intraocular infection. This study suggests that intravitreal vancomycin and ceftazidime is effective against *Nocardia*.

In this case, the patient was secondarily immunocompromised due to his prednisone therapy for nephrotic syndrome. The primary clinical presentation of the patient’s nocardiosis was pulmonary involvement, followed by hematogenous spread to eye. Although the pulmonary and ocular abscesses responded to the treatment, the pulmonary lesion continued, developing brain abscesses as a result of treatment with TMP-SMX not being continued for a suitable amount of time. The duration of therapy depends on the location of infection, the patient’s immune status, and therapic response. It is recommended that disseminated nocardiosis involving brain should be treated for one year, and immunocompetent patients should be treated for at least six months. Premature discontinuation of therapy may also increase the risk of nocardiosis relapse [7–9].

## Conclusions

Because of its low incidence, knowledge regarding *Nocardia* infections is limited, and therefore these infections are very often not considered within initial diagnosis. Clinicians should be aware of the possibility of *Nocardia* infections in all immunocompromised patients and its tendency to disseminate. The rapid diagnosis of *Nocardia* infections and prolonged treatment of the proper antibiotics may significantly improve the prognosis of this life-threatening infection.

## Abbreviations

CT: Computed tomography
MRI: Magnetic resonance imaging
OCT: Optical coherence tomography
PCR: Polymerase chain reaction
TMP-SMX: Trimethoprim-sulfamethoxazole
